# Randomised controlled trial to compare the diagnostic yield of positron emission tomography CT (PET-CT) TARGETed pleural biopsy versus CT-guided pleural biopsy in suspected pleural malignancy (TARGET trial)

**DOI:** 10.1136/bmjresp-2017-000270

**Published:** 2018-02-19

**Authors:** Duneesha de Fonseka, Wendy Underwood, Louise Stadon, Najib Rahman, Anthony Edey, Chris Rogers, Nick A Maskell

**Affiliations:** 1 Academic Respiratory Unit, University of Bristol, Bristol, UK; 2 Clinical Trials and Evaluation Unit, University of Bristol, Bristol, UK; 3 Academic Respiratory Unit, North Bristol NHS Trust, Bristol, UK; 4 Oxford Centre for Respiratory Medicine and Oxford NIHR Biomedical Research Centre, Churchill Hospital, Oxford, UK; 5 Department of Radiology, North Bristol NHS Trust, Bristol, UK

**Keywords:** mesothelioma, pleural disease, imaging/CT MRI etc

## Abstract

**Introduction:**

Pleural malignancy, particularly malignant pleural mesothelioma (MPM) is increasing in incidence due to the long latency period from exposure to asbestos to development of the disease. MPM can be challenging to diagnose. For patients presenting without a pleural effusion, CT-guided biopsy remains the primary choice of biopsy, but the diagnostic sensitivity of this investigation is 70%–75%. Therefore, a proportion of patients will go on to require further biopsies. If the first biopsy is non-diagnostic, the chances of further non-diagnostic biopsies are high in MPM.

**Methods:**

Target is a multicentre randomised controlled trial, aiming to recruit 78 patients over a 30-month period, from 10 centres in the UK. Patients will be randomised to either the standard arm which is a second CT-guided biopsy, or the interventional arm, a positron emission tomography-CT scan followed by a targeted CT-guided biopsy. Patients will be followed up for 12 months (patients recruited in the last 6 months of recruitment will have 6 months of follow-up). MPM biomarker mesothelin will be checked at baseline, 6 month and 12 month follow-up appointments where patients are able to attend these appointments.

**Ethics and dissemination:**

Ethical approval for this trial was granted by the South West—Exeter research and ethics committee (reference number 15/SW/0156). Results of the trial will be published in a peer-reviewed journal and presented at an international conference.

**Trial registration number:**

ISRCTN14024829; Pre-results.

## Introduction

Pleural malignancy can be of primary pleural origin such as mesothelioma or secondary to spread from other sites, most commonly adenocarcinoma of the pleura. Malignant pleural mesothelioma (MPM) is an aggressive and universally fatal tumour, the incidence of which is still considerable in many parts of the world.^1 2^ In the UK, it currently accounts for 1% of malignant disease, with the vast majority of patients developing mesothelioma as a result of previous asbestos exposure.^3^

Patients who have been exposed to asbestos are also at risk of developing benign pleural thickening. This diagnosis can often only be made after the more serious diagnosis of pleural malignancy has been excluded or the patient has undergone interval CT imaging.

In clinical practice, patients with suspicious pleural thickening require a pleural biopsy to histologically confirm a diagnosis of pleural malignancy and also to identify the type of malignancy. When lack of pleural fluid makes a thoracoscopy difficult or when pleural thickening is the only abnormality on CT, tissue is usually obtained using CT-guided pleural biopsy with a Tru-cut needle.^4^ However, the yield remains low as only one small area of the pleural thickening is biopsied, leading to occasional false-negative biopsy results. This means a proportion of patients may require multiple diagnostic procedures to establish a histological diagnosis.

Diagnostic imaging in pleural malignancy remains a significant challenge and a topic of international debate.^5^ Tumour growth is unlike that of solid tumours due to its circumferential expansion, and hence tumour may conceal itself within areas of pleural thickening. This, along with secondary pleural effusion and atelectasis make precise delineation of the tumour volume and radiological staging difficult. In addition, the appearances of benign pleural thickening and pleural malignancy on CT may be similar, and hence other imaging modalities have been evaluated in order to improve the diagnostic pathway for patients.^6^

Local audit data (unpublished) from the lead trust confirm that only 3 out of 15 (20%) repeat pleural biopsies for suspected pleural malignancy (all later confirmed to be cancer) were positive. This compares to four of six patients (66%) for whom specific funding was obtained to undergo a positron emission tomography (PET)-CT-guided pleural biopsy targeting the area of highest metabolic activity instead of a repeat CT-guided biopsy. All six were subsequently confirmed as having malignancy. This has highlighted a potential role for PET-CT in this group of patients, which warrants further investigation.

PET scanning has proved itself a useful tool in the diagnosis and staging of lung malignancy. It identifies areas of tissue with the highest metabolic turnover by highlighting areas of uptake of the radiolabelled glucose analogue, fluorodeoxyglucose (FDG). Initial results in pleural malignancy have been encouraging,^7–9^ but no studies to date have looked at using this modality to target biopsies.

We hypothesise that targeting the CT-guided biopsy to areas of high FDG uptake on PET may improve the diagnostic yield. This would reduce the number of biopsies required to make a diagnosis (with their associated risks and costs).

Identification of a potential biomarker for mesothelioma is another subject of current research.^10 11^ Soluble mesothelin-related peptide (mesothelin) has the greatest sensitivity and specificity in the diagnosis of mesothelioma compared with a number of other markers such as fibulin-3, osteopontin and megakaryocyte potentiating factor.^12–14^ Despite studies demonstrating an elevated mesothelin level at presentation of patients with MPM, its role in the diagnostic arena is yet to be established. In the TARGET trial, we aim to evaluate if serum mesothelin could contribute towards the diagnostic pathway of pleural malignancy.

This trial is the first to address targeted biopsies in patients with suspected pleural malignancy using PET-CT and evaluate the role of a serum biomarker in the diagnostic pathway of pleural malignancy.

If the trial results indicate that PET-CT is superior to standard CT, it could alter the investigation pathway for patients with suspected pleural malignancy and help to expedite a diagnosis. By doing so, we would expect fewer repeated procedures to establish a diagnosis and hence a reduction in associated risks and costs. In addition, by expediting the diagnosis, more patients may be eligible to receive oncological treatment.

## Methods

### Study outcomes

The primary outcome of the study is pleural malignancy correctly identified on the second biopsy.

Patients will be followed up for 12 months or to the end of the trial (if recruited in the last 6 months of the recruitment period). Patients who have a second non-diagnostic biopsy may have further biopsies during this time via other means which may confirm the diagnosis. Some patients maybe given a clinicoradiological diagnosis of pleural malignancy due to characteristic progressive features on subsequent radiology. These cases are usually discussed in the mesothelioma or lung cancer multidisciplinary team (MDT) meeting and members are in consensus of the diagnosis, prior to classifying the disease as malignancy.

There are a number of secondary outcomes to this study:

1. Total number of invasive procedures (video-assisted thoracic surgery or radiology-guided biopsies) undertaken following randomisation to confirm the diagnosis.
2. Time from randomisation to cancer diagnosis (those not diagnosed with cancer will be censored at last follow-up).
3. Time from randomisation to death (survivors will be censored at last follow-up).
4. Total number of hospital attendances following randomisation to confirm the diagnosis.
5. Procedure-related adverse events.
6. Uptake of chemotherapy following a positive diagnosis, in the 12 months following recruitment. Serum mesothelin levels measured at baseline, 6 and 12 month follow-up visits for those followed up for 12 months.
7. PET scan parameters (total glycolytic volume (TGV), maximum and mean standard uptake value (SUV)) (PET-CT group only).
8. Estimated costs associated with health-related resource use from randomisation to diagnosis.

At the end of the study, the biopsies and radiology results will be reviewed by an independent adjudication committee. The committee will be blinded to the results.

### Study overview

The trial is funded by the National Institute for Health Research, research for patient benefit funding stream. The lead centre for the trial is North Bristol National Health Service Trust (NBT) and the study is sponsored by the research and innovation department at NBT.

### Study design

The TARGET trial is a UK-based multicentre parallel group randomised controlled trial, aiming to evaluate whether a PET-CT targeted CT-guided biopsy is superior to a standard CT-guided biopsy in patients with suspected pleural malignancy who have undergone one non-diagnostic biopsy.

### Participant identification

Patients will be identified via the local lung cancer and mesothelioma MDTs. Patients suspected of a pleural malignancy usually have a biopsy and are discussed at the MDT meeting. Therefore, patients with a non-diagnostic biopsy could be identified and screened through the MDT.

### Prescreening, screening and recruitment

Eligible potential patients will be given a patient information leaflet. Provided they are happy to participate in the trial they will be asked to consent to the trial and recruited. Following a baseline assessment gathering data on their demographics, investigations to date, they will be randomised either to the standard arm of the trial or a PET-CT scan followed by a CT-guided biopsy to an area identified on the PET-CT scan.

### Eligibility criteria

#### Inclusion criteria

Patients are eligible if they meet all the criteria below:

- Pleural thickening on CT suspicious for pleural malignancy.
- Have had any form of pleural biopsy in the last 12 months (either by thoracoscopy or under radiological guidance) which was non-diagnostic for cancer.
- Lung cancer/mesothelioma MDT decision to perform a further CT-guided biopsy to pursue a diagnosis.

#### Exclusion criteria

Patients may not enter the study if they meet any of the criteria below:

- Unsuitable for a CT-guided biopsy—inability to cooperate, lie still for the duration of the biopsy, uncorrectable coagulopathy, inability to tolerate a pneumothorax, severe underlying lung disease (patients with a forced expiratory volume in 1 s <35% assessed using simple spirometry).
- Unable to give written informed consent.
- Pregnancy or lactation.
- Age <18 years.
- Pleural thickening not amenable to a radiologically guided biopsy.
- Talc pleurodesis in the previous 6 months.

### Randomisation and blinding procedures

Patients will be allocated on a 1:1 basis to either the intervention (PET-CT prior to CT-guided biopsy) or comparator (CT-guided biopsy only) arm. The allocation will be blocked using varying block sizes and stratified according to enrolling centre. Only authorised personnel will be given access to randomise patients and access will be password protected.

Concealed randomisation will rule out selection bias. The sequence of random allocations will be generated by computer and will be concealed from all clinical and research personnel until a participant has been recruited.

Due to the nature of the investigations performed, participants or investigators will not be blinded to allocation.

#### Research procedures

All patients will have a baseline assessment at the time of their recruitment to the trial. Patients will have blood tests which include a full blood count, urea and electrolytes, a clotting screen and a trial-specific blood test for mesothelin at their baseline assessment. All mesothelin blood tests are analysed at the lead centre NBT. Other centres will send their samples securely to the NBT laboratory.

Patients will have simple spirometry at their baseline assessment to ensure they can tolerate a pneumothorax in the unlikely event this was a complication of the CT-guided biopsy. Patients are randomised at the end of their baseline assessment.

**Figure 1:**
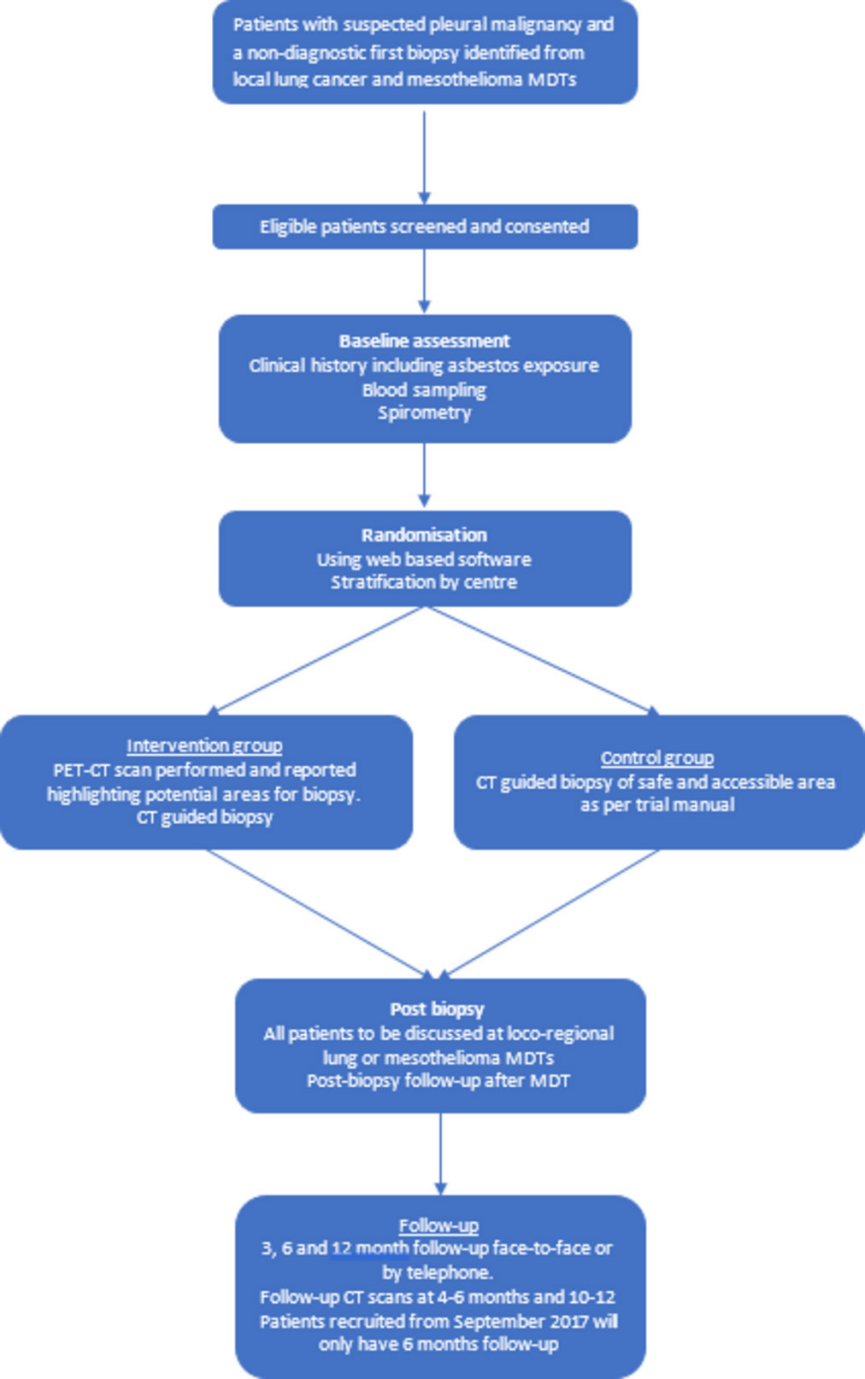
Trial flow chart.

Those randomised to the PET-CT arm will have the PET-CT scan followed by a CT-guided biopsy, ideally with a 2-week period from randomisation. Patients in the standard arm will go straight to a CT-guided biopsy (figure 1).

The mesothelin blood test is trial specific and will be checked at baseline, 6 and 12 month follow-up visits on patients who are able to attend these follow-up visits.

Patients will also have a CT scan between 4 and 6 months and another at 10–12 months. This is of particular importance for those with a non-diagnostic biopsy as a part of the trial, as the expectation is an underlying malignancy that is yet to be diagnosed would become apparent during this follow-up.

#### Follow-up visits

The first follow-up visit will take place when participants attend clinic for their biopsy results. This is usually 1–2 weeks after their biopsy. Adverse events relating to the PET-CT scan and CT-guided biopsy will be captured at this visit. In addition, information regarding their final diagnosis and any further interventions undergone will also be captured.

Three further follow-up visits will occur at 3, 6 and 12 months from randomisation (figure 1). Patients recruited in the last 6 months of recruitment will only have 6 months of follow-up. At each follow-up visit, information regarding any further interventions patients have undergone, adverse events they have experienced and information regarding treatment received will be captured.

### Statistical analysis

An intention to treat analysis will include all randomised participants unless consent to use data is withdrawn. Study protocol adherence and reasons for deviation will be described. Recruitment and participant flow will be described using a Consolidated Standards of Reporting Trials flow diagram. If the number of withdrawals differs by group, the sensitivity of the findings to this attrition bias will be explored.

The number of positive biopsies will be compared, as a proportion of the participants recruited and as a proportion of those with a confirmed diagnosis of mesothelioma on second biopsy using logistic regression. Other binary outcomes will be analysed similarly. Time-to-event outcomes (eg, time to a confirmed diagnosis of mesothelioma) will be compared using survival methods.

Differences between groups will be quantified and reported with 95% CI. If the data are sufficient to allow parameter estimation, we will adjust for centre as a random effect.

The ability of the serum mesothelin levels to predict a positive diagnosis (sensitivity, specificity, positive and negative predictive values, area under receiver operating curve) will be assessed for the study cohort as a whole. Similar analyses of the value of the PET scan parameters to predict a positive diagnosis will be restricted to participants in the PET-CT group.

### Safety reporting

Standard definitions and clinical judgement will be used when reporting any adverse events relating to the trial. As the only research intervention in this trial is a PET-CT scan, significant events are not expected. A list of expected adverse events relating to the PET-CT scan and CT-guided biopsy are listed in the protocol.

## Discussion

Pleural malignancy, particularly MPM can be a challenging disease to diagnose. The heterogeneity of the disease and the tumour itself, the difficulty of establishing the diagnosis on pathology and its slow indolent presentation are some reasons why MPM can be a diagnostic conundrum. A lack of diagnosis can disadvantage the patient by delays in oncological treatment or stopping them from entry into clinical trials.

The role of PET-CT in MPM is still not firmly established although the literature does suggest some use, particularly where patients are considered for surgery, a PET-CT scan can be useful to exclude distant metastases. Given that PET-CT scans can reliably highlight areas of increased metabolic activity, this would be a useful method of targeting biopsies to confirm the diagnosis of pleural malignancy.

If the trial confirms superiority of PET-CT targeted biopsies, this could potentially minimise the number of invasive investigations this cohort of patients are currently subjected to. In addition to the obvious patient benefits it is also likely to have a health economic benefit.

Analysis of FDG uptake indices on PET-CT such as TGV and SUV may provide further information to negate the suspicion of pleural malignancy in certain cases, whereby preventing patients from undergoing unnecessary investigation. Furthermore, identification of distant metastases would provide prognostic information and suitability for certain treatment options. These are some of the secondary benefits of PET-CT scans we would explore as a part of the trial.

Finally, the biomarker serum mesothelin in this cohort may be diagnostically useful in some of the patients in conjunction with the biopsy and cytology result. Change in mesothelin levels over the follow-up period may also be of benefit to the treating physician with their further management of the patient.

### Trial status

The trial opened to recruitment at the lead centre North Bristol NHS Trust in September 2015. Currently, 10 centres are open to recruitment in the UK. Trial will complete recruitment in March 2018.

### Ethical amendments

The protocol is currently in its seventh version, which is published here. All amendments to the initial protocol and the ethics approval dates are shown in table 1.

**Table 1:** Amendments to date

Amendment number	Previous protocol version	Previous date	New protocol version	New date	Summary of change	Date of ethical approval
1	1.0	12 May 2015	Not applicable	Not applicable	No changes to the protocol. Changes to the patient information sheet (PIS) to reflect changes requested by Administration of Radioactive Substances Advisory Committee to change wording and clarification when the fluorodeoxyglucose would be out of the system.	14 September 2015
2	1.0	12 May 2015	3.0	14 January 2016	A previously planned MRI element to the trial was removed. The patient consent form will be uploaded into the National Health Service secure database. Version 2.0 was submitted to the Research Ethics Committee (REC) on the 23 December 2015. However, before this version was approved by the REC, additional changes were made to the protocol and resubmitted as version 3.0 dated 15 December 2016.	02 February 2016
3	3.0	14 January 2016	4.0	23 May 2016	Exclusion criteria changed from ‘pleural thickening not amenable to Tru cut biopsy’ to ‘pleural thickening not amenable to radiologically guided biopsy’ If as a direct result of the positron emission tomography (PET) results, the radiologist feels a pleural biopsy is no longer possible and/or another more easily and safely accessible area has been identified as a result of the PET, then this area should be biopsied instead. Expected serious adverse events relating to disease progression were added to the protocol.	21 June/2016
4	4.0	23 May 2016	5.0	10 October 2016	Addition of new sites Before this amendment could be reviewed by the Ethics committee, we submitted another amendment (see below); they were both reviewed together and approved on 02 December 2016	Not applicable
5	5.0	10 October 2016	6.0	28 October 2016	Change to inclusion criterion ‘any form of pleural biopsy in the previous 6 months’ to ‘any form of pleural biopsy in the previous 12 months’. Change to exclusion criterion ‘prior Talc pleurodesis’ has been changed to ‘Talc pleurodesis in the previous 6 months’	02 December 2016
5	6.0	17 August 2017	7.0	17 August 2017	To increase the recruitment period by 6 months. Patients that were randomised into the study before 4 September 2017 will be followed up for 12 months. Patients randomised after that date will be followed up until 3 September 2018—close of study.	11 September 2017
